# Mycobacteremia from Crushed Hydromorphone Tablet Injection

**DOI:** 10.7759/cureus.1883

**Published:** 2017-11-27

**Authors:** Joseph M Banno, Katherine Shield, Krunal Patel, Anupam A Sule

**Affiliations:** 1 Internal Medicine, St. Joseph Mercy Oakland; 2 St. Joseph Mercy Oakland

**Keywords:** opioid abuse, narcotic injection, mycobacterium abscessus, hydromorphone, mycobacterium chelonae, addiction, mycobacteremia, dialysis contamination

## Abstract

Mycobacterium abscessus is a fast growing, non-tubercular mycobacterium (NTM) found in water. NTM bacteremia is usually seen in immunocompromised patients who have intravascular catheters. Mycobacterium abscessus bacteremia is often caused by exposure to contaminated water supply in hemodialysis units. A 28-year-old female with poorly controlled diabetes mellitus, end stage renal disease (on hemodialysis via Ash catheter), and recurrent deep vein thrombosis presented to the hospital with proximal right leg deep vein thrombosis. On day nine of admission, the patient spiked a fever. Blood cultures revealed Mycobacterium abscessus bacteremia. The patient was observed to be crushing oral hydromorphone and injecting it into her ash catheter using needles retrieved from the sharps disposal container in her room. Although the Michigan Automated Prescription System (MAPS) report for narcotic abuse was unremarkable, a thorough review of her electronic medical chart revealed multiple hospitalizations with drug seeking behavior. This is the first reported case of crushed oral opioid injection resulting in mycobacteremia. Injecting crushed opioids has become more prevalent. Many opioid abusers resort to injecting them in order to achieve the psychotropic effects quicker. A heightened awareness about nontraditional modes of prescription drug abuse and surveillance by prescribers is necessary. This case raises an ethical question: should such patients be prescribed narcotics in the future, as the outcome of injecting oral medications can be fatal.

## Introduction

Non-tubercular mycobacteria (NTM) are usually found as contaminants in water and soil, but have been reported as medication and medical devices contaminants [1-2]. NTM infections often occur in immunocompromised patients who have intravascular catheters. Results of the first national survey of NTM from 1981-1983 showed an annual prevalence of 1.78 NTM cases per 100,000 [3]. Recent studies based on skin testing demonstrate that one in six Americans are now exposed to NTM, as opposed to one in nine nearly 30 years ago [4]. Mycobacterium abscessus gained the reputation of being the most virulent and therapy-resistant member of the rapidly growing NTM group. Healthcare-associated infections caused by this bacterium commonly include skin and soft tissue infections. (It was originally isolated from an abscess, hence the species name). It is also a cause of serious lung infections in persons with various chronic lung diseases, such as cystic fibrosis [5]. Mycobacterium abscessus bacteremia is usually caused by exposure to contaminated water supply in hemodialysis units, possibly due to improper sanitization of equipment [6]. Mycobacterium abscessus and Mycobacterium chelonae were identified as distinct species in 1992. However, they are still mistaken for one another, which is attributed to the sharing of the 16s-ribosomal unit in both species [7].

## Case presentation

A 28-year-old female with a past medical history of end-stage renal disease on hemodialysis and recurrent deep vein thrombosis presented to the emergency room with right leg swelling. The swelling had started after her dialysis session (via her right femoral ash catheter) on the day prior to presentation. Thereafter, she had noticed progressively worsening lower leg pain. She denied any shortness of breath, chest pain, or palpitations. A venous duplex revealed deep vein thrombosis in the right deep femoral vein. The patient was started on intravenous heparin and admitted. The patient had a past medical history of hypertension, subarachnoid hemorrhage, type one diabetes mellitus, seizures, and hypothyroidism. She was taking amlodipine, insulin, isosorbide mononitrate, labetalol, levetiracetam, levothyroxine, pantoprazole, phenytoin, and sevelamer. During a prior admission, she had been diagnosed with superior vena cava syndrome secondary to sclerosis from injecting crushed tablets of hydromorphone into her subclavian Ash catheter. The patient had a history of numerous hospitalizations at multiple health systems for various complaints and had been noted to demonstrate opioid-seeking behavior. The Michigan Automated Prescription System (MAPS) report for opioid prescriptions was unremarkable due to poor outpatient follow-up and illegal procurement of opioids. The patient refused medications intermittently, including warfarin, resulting in a prolonged hospital stay while waiting for therapeutic international normalized ratio (INR) levels. On the ninth day of admission, the patient spiked a fever of 101.8 F. Other key objective findings included a pulse rate of 93 beats per minute, blood pressure of 179/123, respirations of 18 per minute, and oxygen saturation of 99% on room air. The patient was alert and oriented. The lungs were clear to auscultation bilaterally. Cardiac auscultation did not reveal any new murmurs. Her abdomen was soft, non-tender, with normal bowel sounds. No warmth or redness was noted at the site of insertion or distal to the right femoral Ash catheter. On the same day, hospital staff observed the patient injecting crushed hydromorphone tablet in her right groin ash catheter. The patient admitted to holding her oral hydromorphone tablet in her mouth and later crushing it using tap water in a medication cup. She was retrieving needles from the sharp waste disposal container and injecting herself. Blood cultures were collected and she was started on intravenous vancomycin, due to prior history of MRSA bacteremia. The patient remained febrile, and meropenem was started the next day. Blood cultures were repeated on the 13th day of admission. On the 20th day of admission, the repeat cultures isolated vancomycin-resistant enterococcus faecalis. Vancomycin was changed to linezolid, and meropenem was continued. The Ash catheter was removed and the tip culture confirmed vancomycin-resistant Enterococcus faecalis (VRE). On the same day, initial blood cultures revealed gram variable pleomorphic partially acid-fast bacilli. Due to the quick rate of growth, the cultures were sent to the department of community health and were identified as Mycobacterium chelonae (abscessus subtype) on the 22nd day. On the 27th day of admission, amikacin and clarithromycin were added to the antibiotic regimen based on empiric choice of antibiotics to treat Mycobacterium chelonae. On the 33rd day, the isolate was changed to Mycobacterium abscessus. Although the change in species would have raised the possibility of antibiotic failure due to increased macrolide resistance in abscessus compared to chelonae, the amikacin would have treated both adequately. The patient was discharged home with a 42-day regimen of clarithromycin, linezolid, and amikacin based on the susceptibility reports. Outpatient follow-up was not established; however, upon review of electronic medical records, the patient continued to have recurrent admissions for drug seeking behavior.

## Discussion

Patients tampering with opioid medications is an emerging epidemic, with a trivial Google search revealing millions of results instructing users on how to crush and inject oral opioids. An estimated 2.1 million people in the United States abuse prescription opioids [8]. Abusers who crush and inject opioids have twice the risk of dying or developing major complications. The complications associated with this risky pattern of abuse are underreported in the literature. When tap water is used for preparing the injection solution there is a possibility of developing NTM bacteremia. Rapidly growing NTM are resistant to traditional antitubercular drugs, with one study finding 24 different NTM strains that are completely resistant to isoniazid [9]. They may need a multidrug regimen according to the Infectious Disease Society of America (IDSA) guidelines, because monotherapy carries a significant risk of resistance. Studies have identified a gene called 'erm gene' that causes resistance against macrolides in Mycobacteria abscessus, but it is absent in Mycobacteria chelonae [10]. Therefore, it is imperative to identify the correct species to optimize therapy. However, amikacin and tigecycline might be a good empiric choice given the high susceptibility in both Mycobacteria abscessus and chelonae.

## Conclusions

Injecting crushed narcotics has become more prevalent, and a heightened awareness about nontraditional modes of prescription drug abuse is necessary. This case points to the possibility of a rising number of NTM bacteremia cases with increasing prevalence of injection of crushed oral opioid formulations. Treatment of such a patient presenting with persistent fever not responding to antibiotics may require coverage for rapidly growing NTM with amikacin and tigecycline containing regimens.
